# Duodenal Tumor Presenting as Acquired Hemophilia in an 88-Year-Old Woman: A Clinical Case and Review of the Literature

**DOI:** 10.1155/2012/203801

**Published:** 2012-08-26

**Authors:** Nigel P. Murray, Juan Carlos Moncada, Marcelo Moran

**Affiliations:** ^1^Facultad de Medicina, Universidad Mayor, Renato Sánchez 4369, Las Condes, Santiago 27550224, Chile; ^2^Sección de Hematología, Hospital de Carabineros de Chile, Simón Bolívar 2200, Ñuñoa, Santiago 77700199, Chile; ^3^Instituto de Bio-Oncología, Universidad Mayor, Avda. Salvador 95 Oficina 513, Providencia, Santiago 7500710, Chile; ^4^Servicio de Medicina, Hospital de Carabineros de Chile, Simón Bolívar 2200, Ñuñoa, Santiago 7770199, Chile; ^5^Servicio de Cirugía Endoscópica, Hospital de Carabineros de Chile, Simón Bolívar 2200, Ñuñoa, Santiago 7770199, Chile

## Abstract

Acquired hemophilia is a rare disease, presenting with severe hemorrhage, we present a case caused by a duodenal tumor, the clinical management, ethical implications, treatment recommendations, and a review of the literature.

## 1. Introduction

Acquired hemophilia is a rare but potentially life-threatening disease, with an abrupt history of hemorrhage in a person without a personal or familial history of coagulopathy [1–3]. Acquired hemophilia is caused by autoantibodies directed against the functional epitope of Factor VIII (FVIII) resulting in the neutralization of FVIII or increased clearance [4–6]. The incidence has been estimated to be between 0.2 and 1.0 cases/million per year with a mortality of between 8% and 44% [7]. The majority of deaths occur in the first few weeks after the clinical presentation as a result of hemorrhage.

The age distribution is biphasic, with a small group aged between 20 and 30 years, more commonly in women as a result of postpartum antibodies, and a larger group between 68 to 80 years with a male predominance. In approximately 50% of case the antibodies are idiopathic or are associated with autoimmune disease, cancer, diabetes mellitus, hepatitis B and C, or skin and respiratory disease [8–12].

We present the case of an 88-year-old woman who presenting with generalized bruising, vaginal bleeding, and later with melaena.

## 2. Clinical Case

An 88-year spinster presented with generalized bruising and vaginal bleeding, without a personal or family history of bleeding disorders. She had undergone cholecystectomy in 1991, a fractured hip operated with a complete hip replacement in 2007 without hemorrhagic complications. In March of 2010 she suffered an ischaemic stroke, one month later a magnetic resonance showed a small hemorrhagic area in the opposite hemisphere. The Activated Partial Thromboplastin Time was slightly prolonged at this time, 41 seconds with a control of up to 39 seconds, but she underwent a gastrostomy for her neurogenic dysphagia without complications and underwent a rehabilitation program for her stroke. Secondary prevention was started with atorvastatin 40 mg/day, enalapril 20 mg/day, amiodarone 200 mg for atrial fibrillation, aspirin 100 mg/day, and enoxaprin 5000 units/day sc as prophilaxis against DVT.

In May 2010 she presented to the Medical Services, Hospital de Carabineros with the previously described symptoms. Her initial blood tests showed a severe anemia, hemoglobin 5.3 gr/dL, a platelet count of 338,000, reticulocytes 4.5%, an INR of 1.1, and an APTT of 67 seconds (control 28 seconds). A bleeding disorder of the intrinsic pathway was diagnosed. Mixing experiments with a patient plasma: normal plasma of 1 : 1 and 1 : 4 were carried out, showing a correction of the abnormal APTT and thus suggestive of a deficient state. She was initially treated with red cell concentrates and cryoprecipitate. However, the use of cryoprecipiate failed to correct the abnormal APTT.

**Figure 1:**
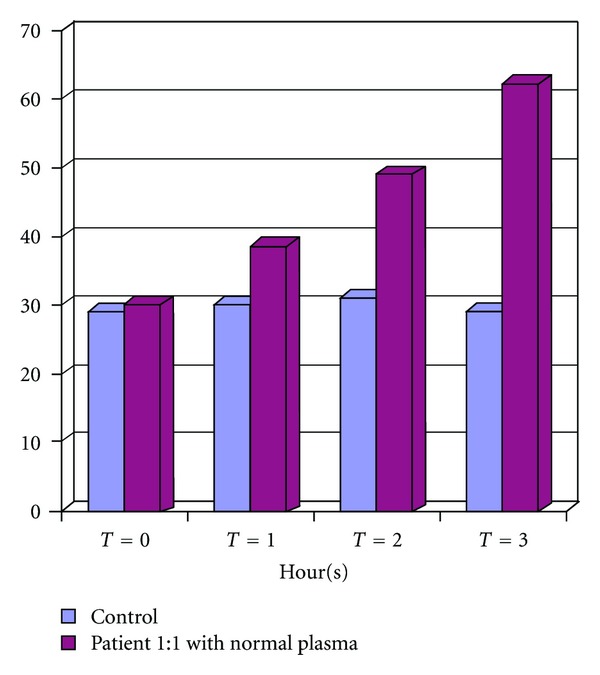
APTT with time in control and patients plasma mixed 1 : 1 with normal plasma.

Studies of factor levels showed a Factor VIII of 6% (normal range 54–151%), Factor IX 72% (normal range 51–137%), Factor XI 54% (normal range 59–151%), Factor von Willebrand 136% (normal range 54–150%), Ristocetin CoFactor 143% (normal 70–150%), and bleeding time of 3 minutes 30 seconds (normal range 2–8 minutes). In view of clinical picture, mixing experiments were repeated with a preincubation of up to 3 hours with normal plasma, and samples taken at 0, 1, 2, and 3 hours with the respective controls (Figure 1), which showed the presence of an inhibitor at 2 and 3 hours. Samples showed an inhibitor anti-FVIII of 32 UI Bethesda (high level).

Immunosuppresive therapy was started with prednisone 1 mg/kg/day, which after 5 days and no change in the APTT was increased to 2 mg/kg/day and combined with cyclophosphamide at a dose of 1.5 mg/kg/day. Support with cryoprecipitate was continued and packed red cells as needed. After three weeks of treatment there was a none significant decrease in the level of anti-FVIII to 24 IU Bethesda but the FVIII level had decreased to 1%.

**Figure 2:**
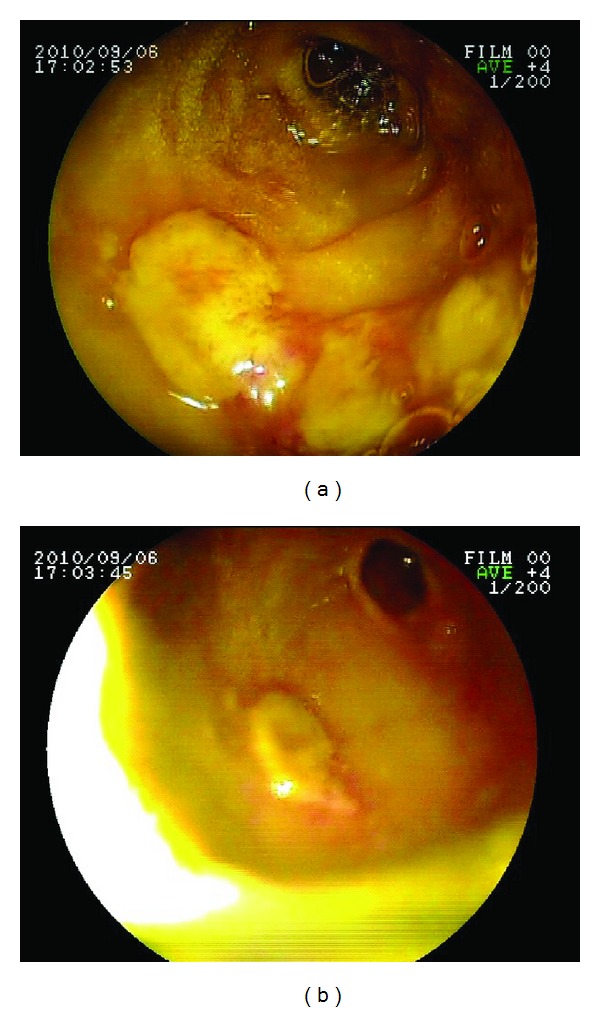
Endoscopic vision of the duodenal tumor.

Transfusions achieved a decrease in the hemorrhages but did not alter the APTT, however the patient then had 2 episodes of melaena with a decrease in the hemoglobin to 7 gr/dL. Upper GI endoscopy was carried out which revealed a duodenal tumor (Figures 2(a) and 2(b)), which appeared to be neoplastic, without active hemorrhage, however a biopsy was not taken for the risk of severe bleeding. An abdominal CT scan did not show the presence of metastasis.

For the high risk a surgical option was considered impossible, the ASA rating was 4. For the patient's associated illnesses, and after consulting the family, the case was presented to the Hospital Ethical Committee. In view of the patient's comorbidities and probable underlying malignancy a decision to stop active treatment was taken. For this reason the use of Factor VIIa or rituximab was not considered a treatment option. The patient died 24 hours later.

In total the patient was transfused, 24 units of packed red cells, 7 units of fresh frozen plasma, and 372 units of cryoprecipitate.

## 3. Discussion

Acquired hemophilia is generally considered as syndrome that is associated with a high mortality rate and a high cost disease. The severity of the clinical manifestations is related to the hemorrhagic episodes, which frequently involved skin, muscles, and soft tissues. However, the majority of the deaths are caused by the underlying disease or as a result of immunosuppressive therapy. Although, the diagnosis is considered to be easily made using simple coagulation tests, the prolonged APTT, an absence of a family or personal history of coagulation disorders, or previous clinical history of hemorrhages, the treatment is complex and expensive.

During the acute presentation, antihemorrhagic treatment is used, when the antibody titre is low, less than 5 UI Bethesda, high dose of purified FVIII or up to 20 units of cryoprecipitate can be used to neutralize the inhibitor. When there are high-inhibitor levels, the alternatives are therapies which bypass the inhibitor, such as activated FVII in a dose of 90 mcg/kg every 2 to 4 hours until the bleeding is controlled, however it is of high cost, approximately U$2,500 per dose. Although there is evidence that FVIIa controls the hemorrhagic event, there are not controlled studies in the literature [13, 14]. Patients with severe hemorrhage are a challenge for the Transfusion Service, for the quantity of blood products used. In general, the prognosis is worse in those patients which high titers of anti-FVIII, low levels of FVIII, and high transfusional requirements. Without the elimination of the anti-FVIII or bypassing the inhibitor, the possibility of surgery is minimal for the risk of hemorrhage.

Steroids alone or in combination with cyclophosphamide are recommended as initial therapy for acquired hemophilia [15], but the median time for a clinical response is between 7 to 8 weeks [16]. The use of rituximab in combination with steroids and cyclophosphamide has been suggested for patients with high antibody titers [17], or alone in patients with contraindications for steroids or cytotoxic therapy. However the response to rituximab alone is less than with combination therapy [18].

In the presented case it was not possible to treat the underlying disease or a biopsy of the duodenal lesion. In 15% of patients with acquired hemophilia a lymphoma or solid tumor is the underlying cause. In the very elderly with underlying comorbidities, the complexities of treatment are not only clinical but also ethical, with the risk of prolonging the suffering of the patient. The coexistence of thromboembolic cardiovascular disease, as in this case, presents a challenge to the physician, the suspension of antithrombotic or antiplatelet drugs, the possible increased risk of thrombosis with the use of agents which bypass the inhibitor, decreased mobility of the patient, and/or activation of the coagulation by infection, alone or in combination, makes therapeutic decisions more difficult.

However, although there is not a consensus on the optimal treatment to improve haemeostasis and/or eradicate the inhibitor in these patients, there are several therapeutic options to treat this rare but severe disease.
